# Atmospheric Pressure as a Cause of Retention of the Placenta

**Published:** 1890-02

**Authors:** 


ATMOSPHERIC PRESSURE AS A CAUSE OF RETENTION OF THE PLACENTA.—Dr. J. Rodriguez, explains a theory of Dr. F. P. Gavivan, of Durango, with regard to certain cases of retention of placenta by *atmospheric pressure.* He, in such a case, having introduced the hand into the uterus, found it impossible to loosen the borders of the placenta; so, with his fingers he pierced the central part of it, when at once, before he had time to make traction, the after-birth lay loose in his hand. He thinks that when the edges of the placenta adhere too firmly, and traction is made on the cord, a vacuum is formed in the centre; when the air enters, by introducing the finger, the placenta is expelled. Certain cases in Dr. Rodriguez's practice seem to confirm the theory. Some of the members present mentioned analogous cases which might be explained by this theory.—*Gaceta Medica,* City of Mexico.

